# MRI-based radiomics prediction of somatostatin receptor ligand response in acromegaly: an automated pipeline study

**DOI:** 10.1210/jendso/bvag198

**Published:** 2026-08-26

**Authors:** Sabina Ruiz, Roger Mateu-Estivill, Ariadna Izquierdo-Gómez, Miriam López-Máñez, Jesús A Alzate, Gemma Monté-Rubio, Maria de la Iglesia-Vayá, Marta Araujo-Castro, Betina Biagetti, Silvana Sarria-Estrada, Federico Vázquez, Fabiola Romero, Elena Valassi, Victor Navas, Mónica Marazuela, Paloma Puyalto, Manel Puig-Domingo, the ACROMICS/ACROFAST Research group, Manel Puig-Domingo, Elena Valassi, Montserrat Marques-Pamies, Silvia Martínez-Couselo, Cristina Carrato, Joan Gil, Paula de Pedro, Federico Vázquez, Mireia Jordà, Betina Biagetti, Silvana Sarria, Olga Giménez-Palop, Anna Aulinas, Susan M Webb, Marta Hernández, Jordi Ferri, Rocío Villar-Taibo, Ignacio Bernabéu, Marta Araujo-Castro, Eider Pascual, Concepción Blanco, Inmaculada Simón, Roxana Zavala, Andreu Simó-Servat, Gemma Xifra, Isabel Pavón, José Antonio Rosado, Rogelio Garcìa-Centeno, Felicia A Hanzu, Mireia Mora, Nuria Vilarrasa, Fernando Guerrero, Soledad Librizzi, María Calatayud, Paz de Miguel, Cristina Alvarez-Escola, Antonio Picó, Carmen Fajardo-Montañana, Eva Benegas, Alfonso Soto, María-Angeles Galvez, Raúl Luque, Cristina Lama, Edelmiro Menéndez, Esteban Delgado, Guillermo Serra, Miguel Sampedro, Mónica Marazuela

**Affiliations:** Endocrine Unit, Hospital de Vic, Vic 08500, Spain; Comparative Medicine and Bioimage Centre of Catalonia (CMCiB), Germans Trias Research Institute (IGTP), Badalona 08916, Spain; Comparative Medicine and Bioimage Centre of Catalonia (CMCiB), Germans Trias Research Institute (IGTP), Badalona 08916, Spain; Comparative Medicine and Bioimage Centre of Catalonia (CMCiB), Germans Trias Research Institute (IGTP), Badalona 08916, Spain; Biomedical Imaging Unit FISABIO-CIPF, Foundation for the Promotion of Research in Healthcare and Biomedicine (FISABIO), Valencia 46020, Spain; Biomedical Imaging Unit FISABIO-CIPF, Foundation for the Promotion of Research in Healthcare and Biomedicine (FISABIO), Valencia 46020, Spain; Comparative Medicine and Bioimage Centre of Catalonia (CMCiB), Germans Trias Research Institute (IGTP), Badalona 08916, Spain; Biomedical Imaging Unit FISABIO-CIPF, Foundation for the Promotion of Research in Healthcare and Biomedicine (FISABIO), Valencia 46020, Spain; Service of Endocrinology and Nutrition, Ramón y Cajal University Hospital, Madrid 28034, Spain; Service of Endocrinology and Nutrition, Vall d’Hebrón University Hospital, Barcelona 08035, Spain; Neuroradiology Unit, Vall d’Hebrón University Hospital, Barcelona 08035, Spain; Service of Endocrinology and Nutrition, Germans Trias University Hospital, Badalona 08916, Spain; Service of Endocrinology, Hospital Central, Instituto de Previsión Social, Asunción 001531, Paraguay; Service of Endocrinology and Nutrition, Germans Trias University Hospital, Badalona 08916, Spain; Service of Endocrinology and Nutrition, Hospital La Princesa, Madrid 28006, Spain; Service of Endocrinology and Nutrition, Hospital La Princesa, Madrid 28006, Spain; Comparative Medicine and Bioimage Centre of Catalonia (CMCiB), Germans Trias Research Institute (IGTP), Badalona 08916, Spain; Department of Radiology, Germans Trias University Hospital, Badalona 08916, Spain; Department of Medicine, Imaging Diagnostic Area, Universitat Internacional de Catalunya, Sant Cugat del Valles 08195, Spain; Comparative Medicine and Bioimage Centre of Catalonia (CMCiB), Germans Trias Research Institute (IGTP), Badalona 08916, Spain; Service of Endocrinology and Nutrition, Germans Trias University Hospital, Badalona 08916, Spain; Department of Medicine, Universitat Autònoma de Barcelona, Campus de Can Ruti, Badalona 08916, Spain

**Keywords:** radiomics, MRI, somatostatin receptor ligands, response prediction, acromegaly

## Abstract

**Context:**

Radiomics may capture tumor characteristics predicting therapeutic response.

**Objective:**

To develop an MRI-based radiomics pipeline for predicting response to SRLs in acromegaly.

**Methods:**

MRI data from 267 subjects across 3 datasets were used to develop an automated tumor detection and segmentation pipeline (Spanish multicenter acromegaly IGTP cohort, *n* = 81; 2 public datasets, *n* = 186). The 3-stage pipeline comprised: (1) automated tumor detection (YOLOv8), (2) 3D segmentation (SegResNet), and (3) radiomic feature extraction (PyRadiomics) with supervised classification. Treatment-response radiomics included contrast-enhanced T1-weighted (Ce-T1W1) MRI from 49 patients treated with SRL. SRL response was defined as ≥50% IGF-1 reduction or normalization. Eleven machine-learning classifiers were evaluated using stratified repeated *k*-fold cross-validation.

**Results:**

Automated segmentation achieved a Dice coefficient of 0.795 using combined Ce-T1WI and T2-weighted images (T2WI), decreasing to 0.765 in the acromegaly-specific cohort. The best-performing model was a 3-feature logistic regression classifier, with an out-of-fold AUC of 0.798 (95% CI: 0.671-0.918), balanced accuracy of 0.793, *F*1-score of 0.815, and correct classification of 39/49 patients (79.6%). Performance improved as the radiomic feature set was reduced from 5 to 2. The most consistent feature was log-sigma-3-0-mm-3D_glcm_ClusterShade, which appeared in 98% of cross-validation folds and contributed most to the model-derived discriminative signal.

**Conclusion:**

In this multicenter radiomics study of SRL response prediction in acromegaly, an automated pipeline achieved good discriminative performance (AUC = 0.798). These findings highlight both the promise and the current challenges of radiomics-based treatment prediction.

Acromegaly is a chronic systemic disorder caused in most cases by a growth hormone (GH)-secreting pituitary adenoma. Persistent excess of GH and insulin-like growth factor 1 (IGF-1) leads to a substantial cardiometabolic, skeletal, and quality-of-life burden if biochemical control is not achieved (1-3). Although transsphenoidal surgery is the recommended first-line treatment, patients with macroadenomas, invasive tumors, or postoperative residual disease require long-term medical treatment with first-generation somatostatin receptor ligands (fgSRLs), pegvisomant, cabergoline, and/or pasireotide. However, in clinical practice, response to medical therapy is heterogeneous and biochemical control remains difficult to achieve in a relevant proportion of patients despite surgery and modern medical therapies (4). This highlights the need for pretreatment predictors of treatment response able to identify likely responders and nonresponders, supporting treatment selection and follow-up strategies (5-9).

Magnetic resonance imaging (MRI) has long been used to search for imaging biomarkers of treatment response in acromegaly, particularly T2-weighted images (T2WI) signal intensity and related texture patterns. Earlier work showed that hypointense T2 signal is associated with better response to fgSRL after surgical failure (6-8). In addition, quantitative approaches, such as T2 histogram analyses, have been related to biochemical response and tumor shrinkage in patients with newly diagnosed disease (6-8). More recently, however, conventional qualitative MRI markers have shown limitations, including observer dependency, limited reproducibility, and inconsistent performance across real-life therapeutic settings (9).

Radiomics offers a more comprehensive and potentially reproducible approach by converting standard MRI data into a large number of quantitative features that describe image intensity, shape, and texture, thereby capturing tumor heterogeneity not appreciable by visual inspection alone. In pituitary tumors, radiomics has been applied to subtype classification, invasiveness assessment, surgical planning, and outcome prediction (10-13). In acromegaly specifically, radiomic models have already shown promising results for preoperative prediction of tumor consistency, with multicenter validation, and for prediction of proliferative activity and response to radiotherapy, using combined radiomic and clinical models (14-16). A multicenter study also demonstrated that MRI-based radiomics could estimate proliferative activity by predicting the Ki-67 index, supporting the biological relevance of quantitative imaging features in somatotroph tumors (16).

Despite these advances, evidence supporting radiomics as a predictor of medical treatment response in acromegaly remains limited. This gap is clinically important because medical therapy is central in persistent or invasive disease, and current therapeutic decisions still rely mainly on biochemical severity, tumor size, invasiveness, and relatively simple MRI descriptors. In this context, a radiomics-based algorithm derived from a well-characterized acromegaly Spanish cohort could provide a noninvasive tool for precision medicine, allowing improved patient stratification before treatment initiation and potentially optimizing use of costly son sometimes long-term therapies.

Accordingly, the aim of the present study was to develop and evaluate an MRI-based radiomics algorithm in a Spanish cohort of patients with acromegaly to predict response to medical therapy. The working hypothesis was that quantitative radiomic features extracted from pituitary MRI would allow the development of automated segmentation algorithms and the identification of radiomic signatures associated with differential hormonal response to medical treatment. The clinical challenge addressed by this study is not image characterization itself, but the identification of objective pretreatment biomarkers capable of selecting the most appropriate first-line medical therapy for individual patients with acromegaly. Therefore, this imaging information could improve individualized therapeutic decision-making for acromegaly patients.

## Patients and methods

### Study design and overall pipeline

**Figure 1:**
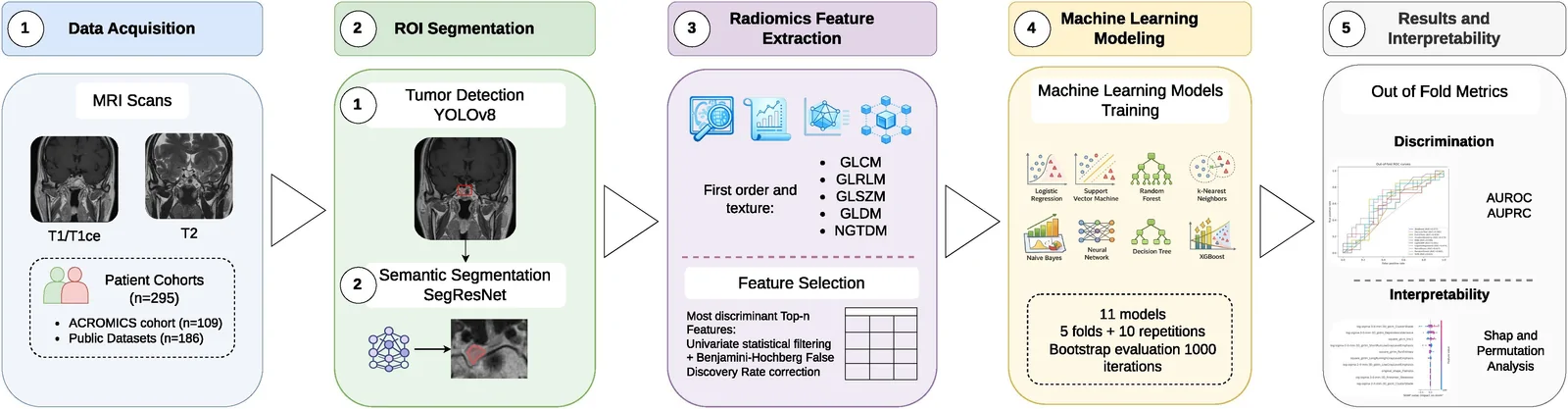
Overview of the automated MRI-based radiomics and machine-learning pipeline. The workflow included MRI preprocessing, YOLOv8-based tumor localization, SegResNet-based 3-dimensional pituitary adenoma segmentation, radiomic feature extraction from manually delineated Ce-T1WI tumor masks, leakage-controlled feature selection within repeated cross-validation, supervised model training, calibration, and model interpretability analyses. The larger multicenter imaging dataset was used for detection and segmentation, whereas treatment-response prediction was restricted to the 49-patient fgSRL radiomics cohort.

The present study proposes a fully automated, end-to-end computational pipeline for the characterization of pituitary adenomas in patients diagnosed with acromegaly, with the ultimate objective of predicting biochemical response to pharmacological treatment (Fig. 1). The pipeline encompasses 3 sequential stages: (1) multisource dataset curation and image preprocessing; (2) automated detection and 3-dimensional segmentation of pituitary adenomas; and (3) radiomic feature extraction and supervised classification of treatment response.

Three independent datasets were used in this study, comprising a total of 267 subjects. Two publicly available datasets were exclusively employed for the development and evaluation of the artificial intelligence (AI)-based segmentation models. These datasets included contrast-enhanced (Ce) T1-weighted images (T1W1) and non-contrast conventional T1WI and T2WI MRI sequences and were distributed in NIfTI format.

The 3 datasets served different analytical purposes. The full 267-subject imaging dataset was used for development and evaluation of the automated detection and segmentation pipeline. In contrast, radiomics-based prediction of treatment response was restricted to the institutional IGTP dataset, because biochemical response data to SRL were available only for this cohort. After applying clinical and imaging eligibility criteria, the final radiomics prediction cohort comprised 49 patients treated with fgSRL.

Manual segmentation masks delineating the pituitary adenoma were available for all subjects and served as ground truth (GT) throughout the study.

#### IGTP dataset

The IGTP dataset was obtained through a multicenter collaboration conducted between 11 tertiary university hospitals in Spain and one tertiary center in Paraguay, participating in the ACROMICS/ACROFAST study. A total of 81 patients with active acromegaly treated with fgSRL were included. All patients gave consent for the analysis of their MRI at the time of the project initiation. Baseline IGF-1 concentrations were recorded before the start of SRL and reassessed at 6 and/or 12 months after treatment onset. Presurgical images were available in 70 patients.

The mean age of the IGTP cohort was 50 ± 1.3 years, and 42 patients (52%) were women. Most patients had undergone pituitary surgery (58, 72%), but for radiomic studies, only presurgical images—mostly obtained at diagnosis—before initiation of fgSRL treatment were used to avoid potential changes in the imaging characteristics related to the postsurgical state or the pharmacologic treatment. Thus, the images used corresponded to a state in which the hormonal activity was high, with a mean IGF-1 level of 243 ± 15% of the upper limit of normal (ULN).

Therapeutic response was classified according to IGF-1 dynamics. Complete response to fgSRL was observed in 27 patients (33.5%), partial response in 14 (17%), and poor response in 40 (49.5%). Response categories were defined based on IGF-1 levels measured in local laboratories and adjusted for age and sex. Partial response was defined as a >50% reduction from baseline IGF-1 without reaching IGF-1 normal value, while poor response was defined as a reduction of <50%. In both cases, responses were assessed after 6 months of treatment, and nonresponder cases required receiving full doses of fgSRL for this period of time (30 mg/month in the case of octreotide and 120 mg/month for lanreotide).

A predefined imaging subgroup was selected for the radiomics analysis. This subgroup included only patients treated with fgSRL who had available pretreatment Ce-T1WI suitable for radiomic feature extraction and complete biochemical response data. This final subgroup comprised 49 patients. To assess potential selection bias, we compared the baseline clinical characteristics of the initial cohort (*n* = 81) with those of the final radiomics cohort (*n* = 49). No clinically meaningful differences were observed in demographic variables, tumor characteristics, or biochemical status, supporting the representativeness of the radiomics cohort and providing no evidence of clinically relevant selection bias. The baseline characteristics of this radiomics cohort are summarized in Table 1. In this radiomics cohort, 26 (53%) patients achieved either a complete or partial response, while 23 (47%) were nonresponders.

**Table 1:** Baseline characteristics of patients included in the final radiomics cohort

	Overall cohort *n* = 49	Responders *n* = 26 (53%)	Nonresponders *n* = 23 (47%)	*P*
Age (mean ± SE)	53 ± 2	55 ± 2	52 ± 3	.46
Female patients	25 (51%)	12 (46%)	13 (57%)	.57
Previous surgery	23 (47%)	12 (46%)	11 (49%)	1
Baseline IGF-1, % ULN (mean ± SE)	279 ± 18	297 ± 28	261 ± 23	.31

The study was conducted in accordance with the ethical principles of the Declaration of Helsinki and implemented and reported in accordance with the International Conference on Harmonized Tripartite Guideline for Good Clinical Practice. The study was approved by the Germans Trias i Pujol Hospital Ethical Committee for Clinical Research (Ref: PI-25-010_radiomica). The protocol and informed consent forms were also approved by the institutional review board of all the participating centers, independent ethics committee, and/or research ethics board of each study site. All patients provided written informed consent to participate in the study.

All images from the participating centers were uploaded to ARIADNA (ARchive of Imaging datA for the Development of New Applications for preclinical and clinical research), an XNAT-based imaging repository hosted at the Centre of Comparative Medicine and Bioimaging of Catalonia (CMCiB) in the IGTP-can Ruti Campus.

#### Public dataset 1—pituitary adenoma MRI dataset

The first publicly available dataset, introduced by Pandit et al (17) and accessible via the Scientific Data repository (DOI: 10.1038/s41597-024-04218-8), was used in our study; it comprises MRI data from a UK pituitary cohort specifically designed to support radiomics and automated segmentation research. It includes high-resolution Ce-T1WI acquisitions from 136 patients with pituitary neuroendocrine tumors of varying histological subtypes—such as somatotroph, lactotroph, corticotroph, gonadotroph, and nonfunctioning adenomas—of which 22 correspond to acromegaly cases. Images were acquired across multiple clinical sites and are provided in NIfTI format. Expert manual segmentation masks are available for each subject, delineating both tumor boundaries and adjacent carotid arteries on the Ce-T1W1 sequence, together with associated clinical, radiological, and pathological metadata. The dataset was released under an open data license and is freely accessible to the research community, making it particularly suitable for the development and cross-institutional validation of automated segmentation methods.

#### Public dataset 2—OpenNeuro pituitary dataset

The second publicly available dataset consists of 50 patients and is hosted on the OpenNeuro platform under the identifier ds006248 (version 1.0.0; (18)). The dataset follows the Brain Imaging Data Structure (BIDS) standard, ensuring compatibility with neuroimaging processing pipelines. Ce-T1WI and T2WI are provided in NIfTI format alongside corresponding manual segmentation masks. This dataset broadens the phenotypic and acquisition-protocol diversity of the training corpus, thereby contributing to the generalizability of the automated segmentation model.

### Manual pituitary adenoma segmentation

Manual tumor segmentation of the IGTP dataset was performed using ARIADNA platform on Ce-T1WI sequences by an experienced imaging analyst, which served as the reference modality for delineation. The remaining sequences (T1WI and T2WI) were spatially aligned to the Ce-T1WI images using a rigid registration with 7 degrees of freedom, ensuring anatomical correspondence across modalities prior to further analysis.

The segmentation process quality control was performed by an expert neuroradiologist, who reviewed, refined, and validated the segmentations to ensure anatomical accuracy and consistency.

Once finalized, the curated segmentation masks were made available within the ARIADNA platform for downstream analysis. These validated annotations were subsequently used by UMIB-IA engineering team (FISABIO-CIPF) to develop and evaluate a deep learning-based automated segmentation model.

### Image preprocessing for automated pituitary adenoma detection

A standardized preprocessing pipeline was applied uniformly to all images across the 3 datasets prior to any subsequent analysis, to minimize inter-site and inter-scanner variability and to ensure spatial consistency.

#### Format standardization and orientation correction

All volumetric images were verified to be in NIfTI format and reoriented to the standard RAS (right–anterior–superior) coordinate convention. This step guarantees consistent anatomical orientation across volumes originating from different acquisition centers, preventing axis-dependent errors in downstream processing.

#### Multimodal registration

For subjects in which T1WI, T2WI, and Ce-T1WI were not inherently co-registered, T1WI and T2WI volumes were registered onto the Ce-T1WI space using the Advanced Normalization Tools Python interface (ANTsPy; (19, 20)). A rigid registration transform was employed to preserve anatomical morphology while correcting for inter-sequence positional discrepancies. Following registration, both modalities were spatially aligned within the same coordinate frame, enabling their joint use as multichannel input for the segmentation model.

#### Intensity normalization

MRI signal intensities are inherently non-standardized across acquisitions performed on different scanners or with varying protocol parameters. To mitigate this confounder, intensity normalization was applied independently to each volume. Specifically, voxel intensity values were rescaled to a common reference range, ensuring that the relative contrast between tissue classes was preserved while absolute intensity levels were harmonized across the dataset.

### Automated pituitary adenoma detection and region-of-interest extraction

#### 2D object detection using YOLOv8

Given the relatively small and variable dimensions of pituitary adenomas within the full brain volume, a preliminary detection stage was incorporated to localize the gland and constrain the subsequent segmentation to a clinically relevant region of interest (ROI). For this purpose, we employed Ultralytics YOLOv8 (21), a state-of-the-art single-stage object detection architecture.

Because all MRI volumes in the dataset were acquired with anisotropic spatial resolution (mean spacing 0.486, 3.3, 0.486), each 3-dimensional volume was decomposed into individual 2-dimensional coronal slices. For each slice, the GT bounding box was derived by computing the minimum axis-aligned rectangle enclosing the corresponding manual segmentation mask. These bounding boxes constituted the supervision signal for YOLOv8 training.

At inference time, the model predicted a bounding box for each coronal slice. The slice associated with the largest predicted bounding box area was identified as the reference slice, as it corresponds to the axial level at which the adenoma achieves its maximum cross-sectional extent. The 3-dimensional ROI was then constructed by propagating the dimensions of this maximal bounding box uniformly across all coronal slices of the volume, yielding a cuboid crop centered on the pituitary region.

The clinical validity of this ROI definition was verified by confirming that 100% of test-set subjects had their complete manual segmentation mask contained within the extracted ROI. This criterion ensures that no GT adenoma voxels were excluded prior to the segmentation stage, which is a prerequisite for unbiased downstream evaluation.

The cropped ROI was extracted identically from both the Ce-T1WI and T2WI volumes, as well as from the corresponding binary segmentation masks, to serve as input for the 3-dimensional segmentation model.

### Automated three-dimensional segmentation

#### Model architecture

Automated volumetric segmentation of the pituitary adenoma was performed using Auto3DSeg, a self-configuring deep learning framework for 3-dimensional medical image segmentation within the MONAI ecosystem (22). Auto3DSeg automatically adapts hyperparameters, patch sampling strategies, and data augmentation schemes based on the input dataset characteristics. For this study, the framework was configured to employ the SegResNet architecture, a residual encoder–decoder network specifically optimized for volumetric biomedical tasks (23).

The segmentation model received as input 2-channel 3-dimensional patches comprising the co-registered and preprocessed Ce-T1WI, T1WI, and T2WI volumes cropped to the adenoma ROI, as described previously. The corresponding binary manual segmentation masks served as the GT supervision signal.

#### Training strategy and cross-validation

Prior to model development, a held-out test consisting of 15% of the total cohort (*n* = 40) was randomly selected and set aside exclusively for final performance evaluation. The remaining 85% of the dataset (*n* = 227) was employed for model training and internal validation via a stratified 5-fold cross-validation scheme, ensuring that each subject appeared in exactly one validation fold across the 5 iterations.

#### Segmentation evaluation metrics

Model performance was quantified using 3 complementary metrics. The Dice similarity coefficient (24) measured the volumetric overlap between predicted and GT masks. The intersection over union (25), also referred to as the Jaccard index, provided an alternative overlap measure with greater sensitivity to false positives. The 95th-percentile Hausdorff distance (26) assessed boundary agreement by computing a robust estimate of the maximum surface-to-surface distance, thereby capturing localized segmentation errors at the adenoma periphery whilst remaining insensitive to outlier voxels.

### Radiomic feature extraction

Radiomic feature extraction was performed only in the IGTP institutional cohort, as this was the only dataset with available hormonal response data to SRL therapy. Although 81 fgSRL-treated (pretreatment IGF-1 levels and treatment response classification) patients were initially eligible, the final prediction cohort was restricted to 49 patients after excluding cases without suitable pretreatment Ce-T1WI MRI, incomplete imaging data, postsurgical-only imaging, images not appropriate for baseline radiomics, processing errors, or radiological uncertainty affecting tumor delineation. To ensure a homogeneous tissue baseline, only pretreatment (presurgical) MRI examinations were considered. Furthermore, patients lacking Ce-T1WI volumes were also excluded. Applying these strict clinical and technical selection criteria resulted in a final radiomic cohort of 49 patients. Feature extraction was performed on the Ce-T1WI volumes using the manually delineated segmentation masks as the ROI to avoid the introduction of any systematic bias attributable to automated segmentation inaccuracies.

Radiomic features were computed using PyRadiomics (27), an open-source Python library conformant with the Image Biomarker Standardization Initiative (IBSI) guidelines (28). The following feature classes were extracted: first-order statistical features, shape-based 3-dimensional descriptors, gray level co-occurrence matrix (GLCM) texture features, gray level run length matrix features, gray level size zone matrix features, neighboring gray tone difference matrix features, and gray level dependence matrix features. Feature extraction was performed on both the original image and on filtered derivatives (Laplacian of Gaussian, wavelet decompositions) to capture multi-scale textural information.

### Radiomic feature selection

Given the high dimensionality of the radiomic feature space relative to the available sample size (*n* = 49), a multistage feature selection pipeline was applied independently within each training fold to mitigate overfitting and reduce the risk of data leakage. All selection steps were performed exclusively on the training data of each fold. First, non-radiomic metadata and diagnostic variables were excluded. For each remaining feature, distributional normality was assessed using the Shapiro–Wilk test (*α* = .05). Normally distributed features were evaluated for group discrimination using an independent Student *t*-test, whereas non-normally distributed features were analyzed using a 2-sided Mann–Whitney *U* test. Raw *P*-values were adjusted for multiple comparisons using the Benjamini–Hochberg false discovery rate (FDR) procedure with a significance threshold of *q* ≤ 0.05, and features were subsequently ranked by their univariate ROC–AUC.

No individual candidate features consistently crossed the FDR-corrected significance threshold within the cross-validation framework. Therefore, to avoid excluding potentially informative and stable texture patterns, feature selection was driven by univariate discriminative ranking rather than by strict FDR filtering alone. To reduce redundancy, a greedy correlation-pruning step was then applied: when 2 features showed a Spearman correlation coefficient |*ρ*| ≥ 0.90, the lower-ranked feature was discarded. Finally, to constrain model complexity according to the sample size available, the number of features entering each classifier was restricted. The main automated analyses focused on configurations retaining 3 and 5 features, which represented parsimonious trade-offs between dimensionality and discriminative performance. An additional configuration applied principal component analysis (PCA) to the 5-feature set, retaining 2 principal components, to assess whether linear dimensionality reduction improved generalization.

Because fold-wise feature selection may be unstable in small, high-dimensional datasets, feature selection stability across folds and repetitions was also monitored. As a post hoc stability-informed sensitivity analysis, features were ranked according to their selection frequency across the repeated cross-validation procedure. The most consistently selected features were then retained as a fixed candidate signature, and model performance was re-evaluated using the same 5-fold, 10-repeat cross-validation framework restricted to this feature subset.

### Treatment response classification

#### Outcome definition

As described above, biochemical response to pharmacological treatment was categorized based on post-treatment serum IGF-1 levels, in accordance with established consensus criteria for acromegaly management and our own data (7, 29), which initially stratifies patients into 3 categories: no response, partial response, or complete response. However, to mitigate the severe class imbalance inherent in the raw clinical distribution and ensure statistical stability during classifier training, these categories collapsed into a binary outcome. Specifically, patients achieving either a partial or a complete biochemical response were pooled into a single “Responders” category, representing individuals who derived a meaningful clinical benefit from the therapy. Conversely, patients with IGF-1 values showing a reduction <50% from their pretreatment basal were classified as “Non-responders.” The cohort comprised 26 responders and 23 nonresponders, reflecting a near-balanced class distribution.

#### Classifier development and validation pipeline

For each treatment arm, a benchmarking suite of 11 supervised machine-learning architectures was developed using Scikit-learn (30): support vector machine (SVM), logistic regression (LR), random forest (RF), extra trees (ET), decision tree (DT), Gaussian naive Bayes, K-nearest neighbors, gradient boosting, adaptive boosting, a multilayer perceptron neural network, and light gradient boosting machine (LightGBM). All base classifiers were embedded within standardized preprocessing pipelines comprising median imputation (SimpleImputer), *z*-score normalization (StandardScaler), and zero/low-variance feature removal (VarianceThreshold), executed strictly and independently within each training partition to prevent any form of data leakage.

To systematically counteract class imbalance, cost-sensitive learning was natively integrated into the pipeline. This was achieved by applying class-weight adjustments (setting the parameter to “balanced” or “balanced_subsample” depending on the architecture) across the SVM, LR, RF, ET, DT, and LightGBM classifiers, thereby penalizing misclassifications of the minority class more heavily during the optimization phase.

Hyperparameter optimization was performed via search loops executed strictly within the inner training folds, using ROC-AUC as the optimization criterion to prevent data leakage. Search spaces targeted core regularization strengths and structural constraints to prevent overfitting. Model performance was estimated using a stratified repeated *k*-fold cross-validation scheme (*k* = 5 folds, 10 repetitions; 50 total evaluation rounds per classifier), ensuring feature selection was reapplied independently within each training partition. Out-of-fold (OOF) predictions were aggregated across all repetitions to produce a single pooled performance estimate. Following cross-validation, the predicted probability outputs of the best-performing classifier were calibrated using Platt scaling (3-fold inner cross-validation). Classifier performance was assessed on OOF predictions using ROC–AUC as the primary metric, alongside standard classification and correlation metrics (accuracy, balanced accuracy, *F*1-score, sensitivity, specificity, PPV, NPV, and MCC), with 95% confidence intervals computed via bootstrapping (*n* = 1000 iterations). Model interpretability and clinical utility were comprehensively evaluated using native coefficients, permutation importance (20 repetitions), SHAP values, LIME local explanations, and decision curve analysis (DCA).

To formally evaluate the robustness of the final model against the well-recognized risk of instability associated with small-sample, high-dimensional prediction modeling, we additionally performed a bootstrap-based model stability analysis on the automated 3-feature LR configuration, following the framework proposed by Riley et al and Riley and Collins (31, 32). The entire model-development procedure, comprising feature selection and model fitting, was repeated on 1000 bootstrap resamples of the 49-patient cohort, drawn with replacement at the patient level and stratified by treatment-response status. For each replicate, the resulting model was applied to the original 49 patients, and its predictions were compared against those of the model developed on the full original sample, yielding a prediction instability plot, a calibration instability plot based on the mean absolute prediction error (MAPE), and a classification instability index (CII) for each patient. To further quantify the optimism arising from benchmarking 11 candidate classifiers and selecting the best-performing architecture, a second bootstrap analysis (1000 replicates) was performed in which the OOF AUC of all 11 classifiers was recalculated on the same resampled patients within each replicate; the AUC of the replicate-specific best-performing classifier was compared with that of the prespecified final classifier (LR) across replicates, and the mean difference between the 2 was taken as an estimate of selection-induced optimism.

To further address model calibration and clinical applicability, in line with recent guidance on performance measures for predictive AI models (33, 34) and the TRIPOD + AI reporting statement (35), we additionally computed calibration-in-the-large, calibration intercept, and calibration slope via logistic recalibration of the OOF predicted probabilities, and performed DCA, reporting net benefit relative to the default “treat-all” and “treat-none” strategies across a range of threshold probabilities. The final model is additionally reported as an explicit equation on the original (non-standardized) radiomic feature scale, together with the imputation values used for missing data, to enable independent application and external validation. A completed TRIPOD + AI checklist is provided as supplemental material (Supplemental Material (35)).

## Results

### Performance of the automated pituitary adenoma segmentation

The SegResNet-based segmentation model showed good performance when evaluated on the public pituitary adenoma dataset, achieving a Dice coefficient of 0.835 using combined Ce-T1WI and T2WI data. When images from the ACROMICS/ACROFAST acromegaly cohort were incorporated, performance decreased, consistent with the greater heterogeneity of real-world multicenter imaging data.

Several preprocessing and standardization strategies were evaluated to improve model generalizability across datasets. However, more intensive standardization did not consistently improve segmentation accuracy, suggesting that excessive normalization may remove clinically relevant image contrast or introduce additional variability. The best performance in the combined multicenter dataset was obtained after incorporating YOLO-based tumor localization before 3-dimensional segmentation. In this configuration, the model achieved a Dice coefficient of 0.795 using combined Ce-T1WI and T2WI, outperforming the Ce-T1WI-only configuration (Dice = 0.777). These findings support the use of a localization-guided, multimodal segmentation strategy for automated pituitary adenoma delineation in heterogeneous multicenter MRI datasets.

### Radiomics

#### Sample

A total of 81 patients from the ACROMICS/ACROFAST institutional cohort with acromegaly and pituitary tumors were initially considered for inclusion in the radiomics analysis. Several exclusion criteria were subsequently applied to ensure data quality, imaging consistency, and reliable tumor characterization. Eleven cases were removed because of image-processing errors. Seven patients were excluded because complete imaging data were unavailable, specifically the required acquisitions (T1WI, T2WI, Ce-T1WI). Nine patients were excluded because only postsurgical images were available. Finally, 5 patients were additionally excluded after radiological assessment because of factors that could compromise accurate analysis, including multiple tumor structures, poor image quality, abnormal anatomy related to previous surgical interventions, or inability to reliably identify the tumor mass. The final radiomics prediction cohort therefore comprised 49 patients treated with fgSRL who had suitable pretreatment Ce-T1WI and biochemical response data. This cohort included 26 responders (53.1%) and 23 nonresponders (46.9%), reflecting a near-balanced class distribution.

#### Feature selection and stability

Following the multistage feature selection pipeline executed independently within each of the 50 cross-validation folds, a single radiomic descriptor emerged with exceptional robustness: log-sigma-3-0-mm-3D_glcm_ClusterShade (gray level co-occurrence matrix ClusterShade computed under a Laplacian of Gaussian filter at a coarse scale of sigma = 3.0 mm). This feature was selected in 49 of 50 folds (98.0%), achieving a median univariate rank of 1.0 and a mean univariate AUC of 0.788 (Table 2).

**Table 2:** Feature selection stability across 50 cross-validation folds (5-fold × 10 repeats)

Feature	Folds selected/50	Frequency	Median rank	Mean AUC
log-sigma-3-0-mm-3D_glcm_ClusterShade	49	0.98	1.0	0.788
log-sigma-2-0-mm-3D_glcm_ClusterShade	32	0.64	3.0	0.748
square_glrlm_RunEntropy	29	0.58	4.0	0.743
square_glrlm_LongRunHighGrayLevelEmphasis	28	0.56	5.0	0.738
log-sigma-3-0-mm-3D_gldm_DependenceVariance	22	0.44	6.0	0.711

Frequency = proportion of folds in which the feature was included in the final model. Median rank = ranking by univariate discriminative power within evaluated folds.

The second most frequent feature, log-sigma-2-0-mm-3D_glcm_ClusterShade, was selected in 32 folds (64.0%) with a median rank of 3.0. The third most frequent feature, square_glrlm_RunEntropy, was selected in 29 folds (58.0%) with a median rank of 4.0. All remaining candidates exhibited selection frequencies below 30.0%. As expected from the high dimensionality of the feature space, no individual radiomic feature crossed the conservative FDR-corrected significance threshold within the training loops. Consequently, feature selection relied on the robust univariate discriminative ranking, which reliably prioritized the highly stable ClusterShade family across the evaluation folds.

#### Classifier performance

**Figure 2:**
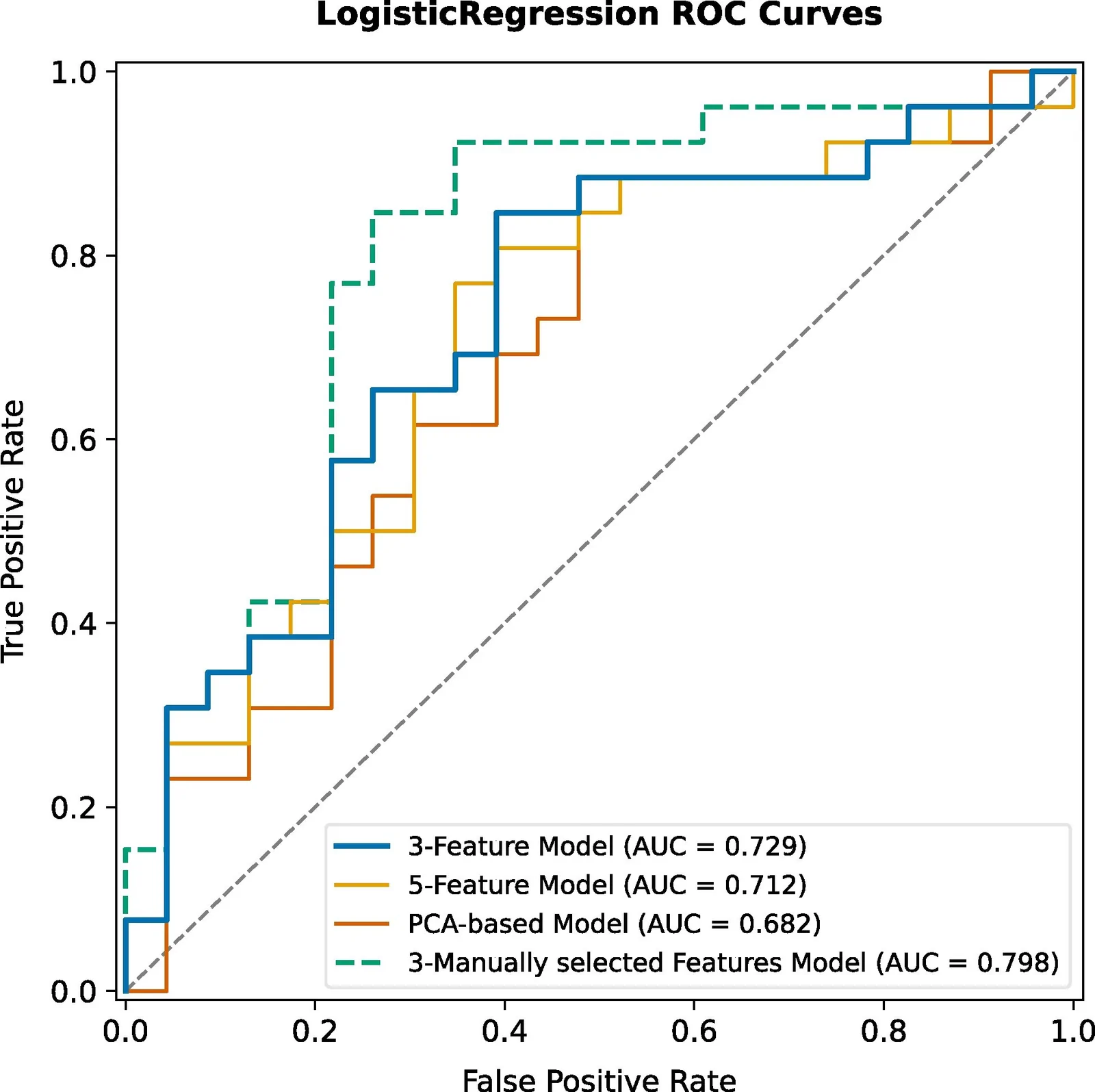
Out-of-fold (5 folds—10 repetitions) receiver operating characteristic (ROC) curves of the logistic regression models for predicting treatment response in pituitary tumors (0 = nonresponse, 1 = partial or complete response). The plot compares the discriminative performance of the 3-feature, 5-feature, PCA-based feature, and 3-manual feature selection approaches.

A comprehensive benchmarking execution involving all 11 supervised architectures was conducted across the various feature-subset sizes and the PCA-reduced variant. A consistent trend emerged across the entire suite of classifiers: classification performance improved as the feature space was streamlined and parsimoniously reduced, reaching its peak when expert post hoc filtering was applied. Among all combinations evaluated, the LR model consistently achieved the highest discriminative power. Table 3 reports the detailed OOF performance for these leading LR configurations, comparing the automated 5-feature, automated 3-feature, PCA-based, and 3-manual feature architectures (Fig. 2).

**Table 3:** Out-of-fold performance of logistic regression across feature-set configurations (*n* = 49)

Configuration	OOF AUC	95% CI	Bal. Acc.	*F*1	MCC	Accuracy
LR—5 features	0.712	[0.558-0.849]	0.653	0.667	0.306	0.653
LR—3 features	0.729	[0.592-0.866]	0.672	0.692	0.344	0.673
LR—5 feat. + PCA (2 components)	0.682	[0.530-0.839]	0.634	0.640	0.267	0.633
LR—3 manual features selection	0.798	[0.671-0.918]	0.793	0.815	0.590	0.796

Abbreviations: AUC, area under the ROC curve; Bal. Acc., balanced accuracy; CI, 95% bootstrap confidence interval (*n* = 1000); MCC, Matthews correlation coefficient.

**Figure 3:**
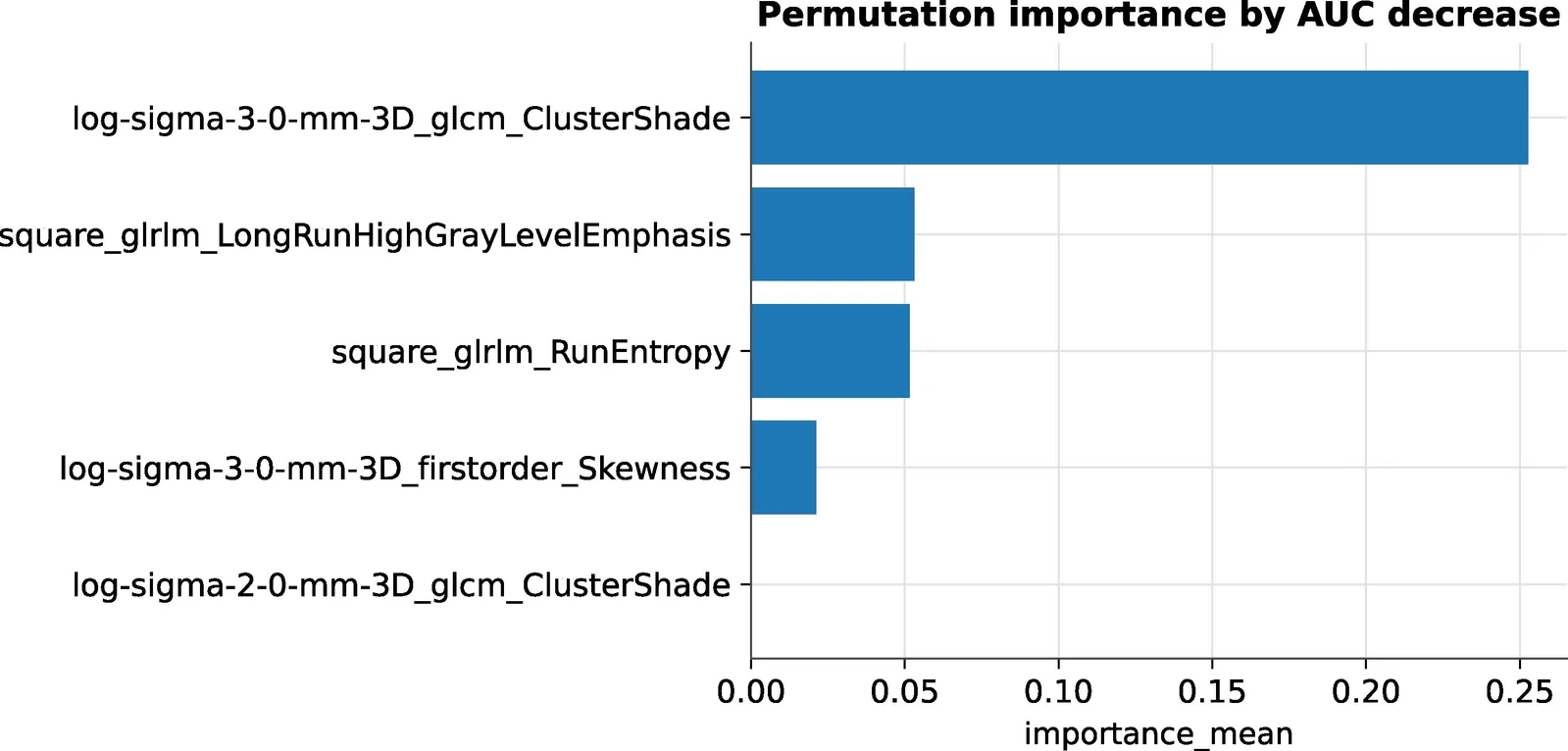
Permutation feature importance analysis based on the mean decrease in the area under the curve (AUC) for the 5-feature model configuration. Model performance is primarily driven by a small subset of texture-based radiomic features, with log-sigma-3-0-mm-3D_glcm_ClusterShade demonstrating the most substantial contribution to predictive accuracy.

**Figure 4:**
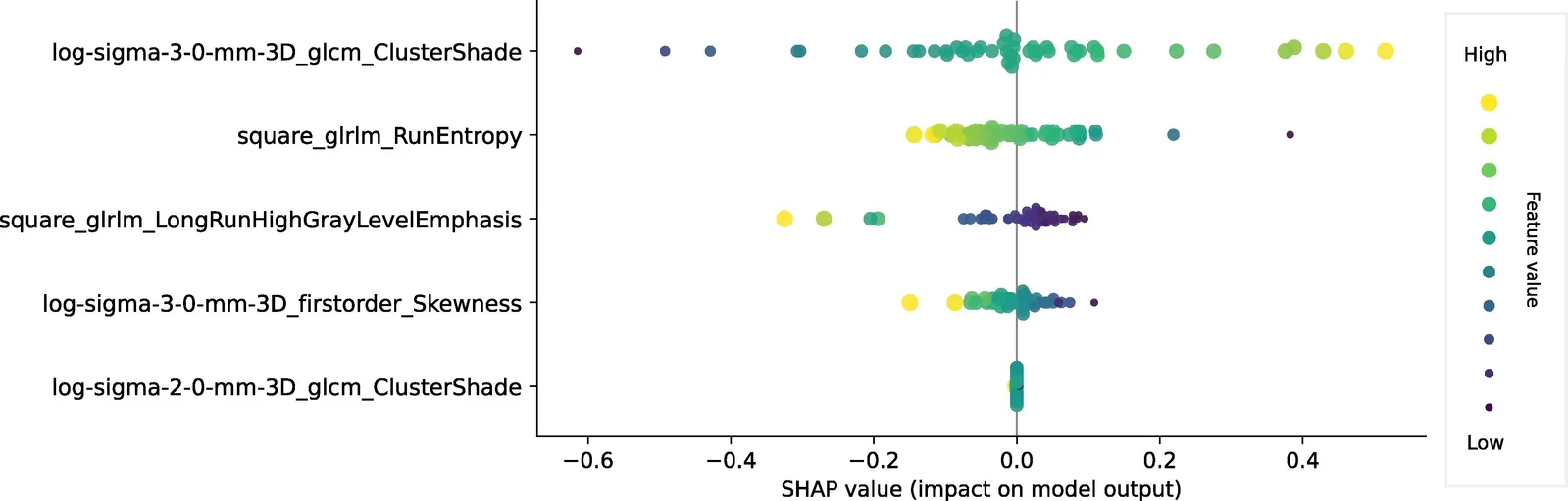
SHAP beeswarm plot illustrates the directional contribution of individual radiomic features to the 5-feature model predictions. Each point represents a patient sample, and point size indicates the corresponding feature value (small points represent lower values and larger points represent higher values). The horizontal position reflects the SHAP value. Positive SHAP values indicate a higher predicted probability of treatment response, highlighted by the positive impact of elevated log-sigma-3-0-mm-3D_glcm_ClusterShade values.

The baseline automated 5-feature model yielded an OOF AUC of 0.712 (95% CI: 0.558-0.849), with a balanced accuracy of 0.653 and an *F*1-score of 0.667. This multifeature framework subsequently served as the foundation for global permutation and SHAP exploratory analyses (Figs. 3 and 4). Streamlining this feature space via automated algorithms into the automated 3-feature configuration resulted in a marginal increment in the discriminative signal, achieving an AUC of 0.729 (95% CI: 0.592-0.866), a balanced accuracy of 0.672, and an overall accuracy of 67.3% (33/49 patients). Conversely, projecting the 5-feature space via PCA to 2 principal components severely degraded the classification metrics, yielding the lowest performance across the benchmark (AUC = 0.682, MCC = 0.267). This outcome confirms that linear projection methods fail to preserve the underlying predictive feature topographies required for response stratification.

**Figure 5:**
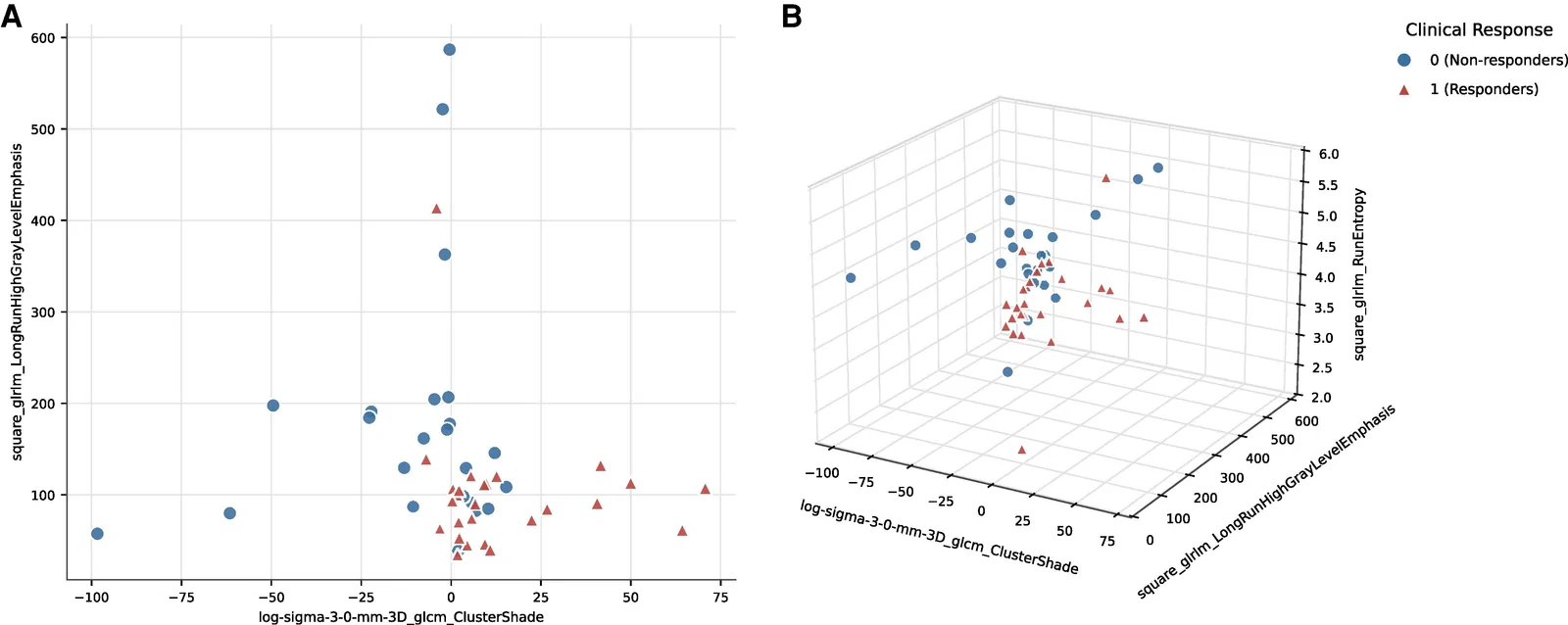
Feature space distribution of selected radiomics features for treatment response prediction in pituitary tumors. (Left panel, A) Two-dimensional scatter plot comparing the 2 primary features: *log-sigma-3-0-mm-3D_glcm_ClusterShade* (x-axis) and *square_glrlm_LongRunHighGrayLevelEmphasis* (y-axis). (Right panel, B) Three-dimensional scatter plot incorporating the third feature, *square_glrlm_RunEntropy* (*z*-axis), to display the spatial separation of the patient cohort. In both plots, marker shapes indicate clinical response status: circles represent responders (1) and triangles represent nonresponders (0).

Crucially, the 3-manual features model, constructed via post hoc expert isolation from the 5-feature candidate pool, demonstrated a statistically substantial improvement over all purely automated algorithmic selection approaches. This optimized expert configuration achieved the peak discriminative performance of the study, with an OOF AUC of 0.798 (95% CI: 0.671-0.918), a balanced accuracy of 0.793, an *F*1-score of 0.815, and a robust Matthews Correlation Coefficient (MCC) of 0.590. This architecture successfully cross-validated an overall classification accuracy of 79.6% (Fig. 5). Furthermore, the primary optimized LR models exhibited highly competitive Brier scores (eg, 0.21 for the 3-feature automated model), and their raw probability outputs were calibrated utilizing Platt scaling to ensure the clinical reliability of the estimation outputs.

#### Model stability and optimism of model selection

**Figure 6:**
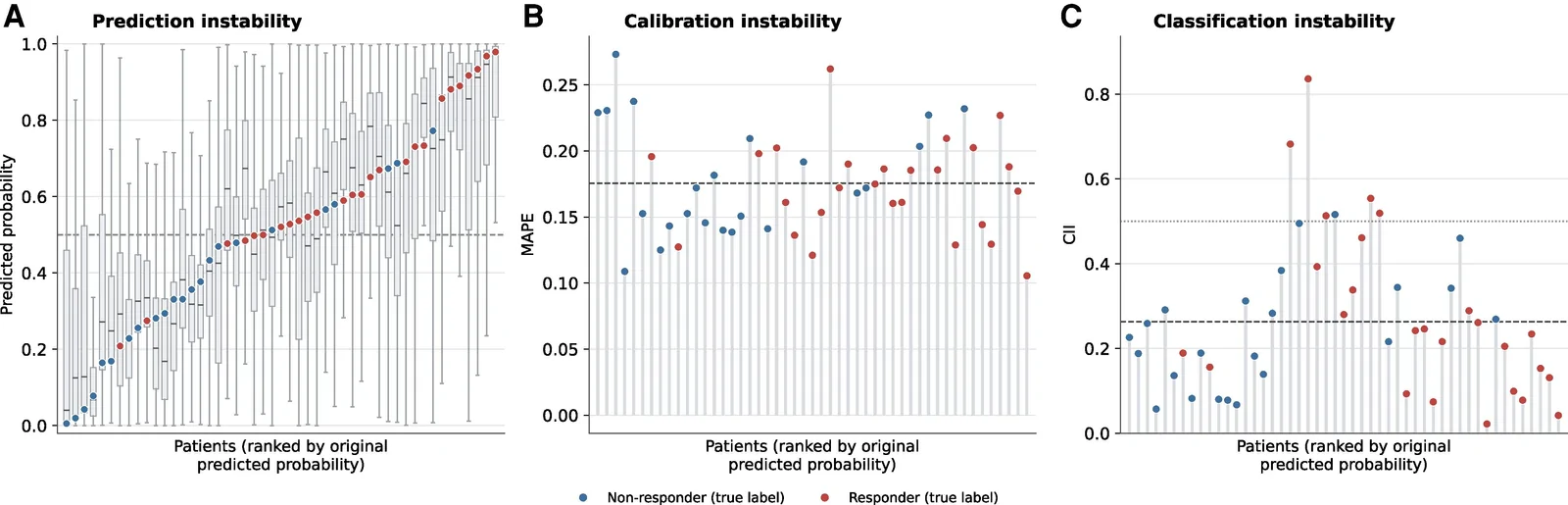
Bootstrap-based stability analysis of the automated 3-feature logistic regression model (*n* = 49 patients, 1000 bootstrap replicates), following Riley and Collins (33, 34). (A) Prediction instability plot: boxplots show the distribution of predicted probabilities for each patient across bootstrap replicates in which the entire model-development procedure was repeated; colored points show the prediction from the model developed on the full sample, and patients are ranked by this original predicted probability. (B) Calibration instability plot: mean absolute prediction error (MAPE) between the original and bootstrap-derived predictions for each patient. (C) Classification instability plot: classification instability index (CII), the proportion of bootstrap models classifying a patient differently from the original model at a 0.5 probability threshold.

Because the automated 3-feature LR model was developed in a small cohort (*n* = 49) relative to the number of candidate predictors and classifiers evaluated, we quantified its stability and the optimism attributable to comparing 11 classifiers using bootstrap-based analyses (1000 replicates each). Across the 1000 replicates, predicted probabilities for a given patient differed from those of the model developed on the full sample by an MAPE of 0.176, and the CII averaged 0.263, indicating that, on average, 26.3% of bootstrap-derived models classified a given patient differently from the original model. For a subset of 6 of 49 patients (12.2%), the majority of bootstrap models produced a classification opposite to that of the published model (Fig. 6). Feature-selection instability was also evident under bootstrap resampling: log-sigma-3-0-mm-3D_glcm_ClusterShade was reselected in 50.5% of replicates, log-sigma-2-0-mm-3D_glcm_ClusterShade in 27.0%, and square_glrlm_RunEntropy in 15.5%, confirming that only the first feature was consistently robust to resampling (Table 4).

**Table 4:** Bootstrap-based stability and model-selection optimism metrics for the automated 3-feature logistic regression model (*n* = 49; 1000 bootstrap replicates)

Metric	Value
Mean absolute prediction error (MAPE), mean	0.176
Classification instability index (CII), mean	0.263
Patients with CII > 0.50	6/49 (12.2%)
Feature reselection frequency—log-sigma-3-0-mm-3D_glcm_ClusterShade	50.5%
Feature reselection frequency—log-sigma-2-0-mm-3D_glcm_ClusterShade	27.0%
Feature reselection frequency—square_glrlm_RunEntropy	15.5%
Winner AUC (best-of-11), mean [95% CI]	0.741 [0.610-0.871]
LogisticRegression AUC (prespecified), mean [95% CI]	0.731 [0.592-0.866]
Selection-induced optimism (ΔAUC)	+0.0095
LogisticRegression selection frequency (best-of-11)	68.0%

**Figure 7:**
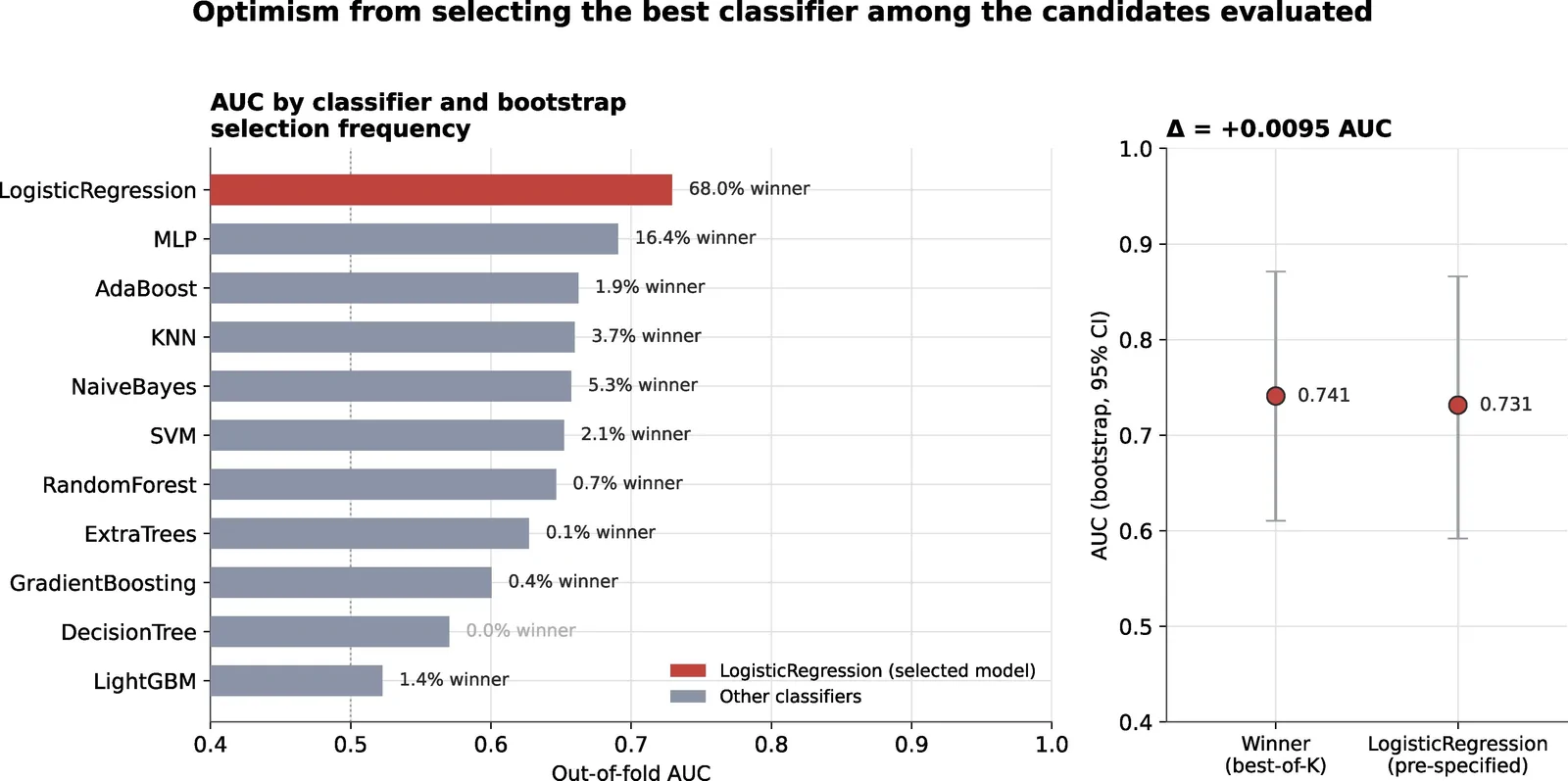
Optimism attributable to selecting the best-performing classifier among the 11 candidates evaluated (1000 bootstrap replicates). (Left panel) Out-of-fold AUC for each classifier, with the percentage of replicates in which each classifier achieved the highest AUC on the same resampled patients. (Right panel) Mean AUC (95% bootstrap confidence interval) of the replicate-specific best-performing classifier (“winner”) compared with the prespecified logistic regression model in the same replicates.

The optimism attributable to selecting the best-performing classifier among the 11 candidates was small: the mean OOF AUC of the replicate-specific “winner” was 0.741 (95% CI: 0.610-0.871), compared with 0.731 (95% CI: 0.592-0.866) for the prespecified LR model in the same replicates, corresponding to an estimated selection-induced optimism of 0.0095 AUC points. Logistic regression was the best performing classifier in 68.0% of the 1000 replicates, indicating that its selection as the final model was not primarily driven by optimistic model selection (Fig. 7, Table 4). These findings should be interpreted together: while the choice of LR over the other 10 classifiers appears robust, the individual-level predictions of the selected model carry nontrivial uncertainty consistent with the limited sample size.

#### Calibration, clinical utility, and the final model equation

**Figure 8:**
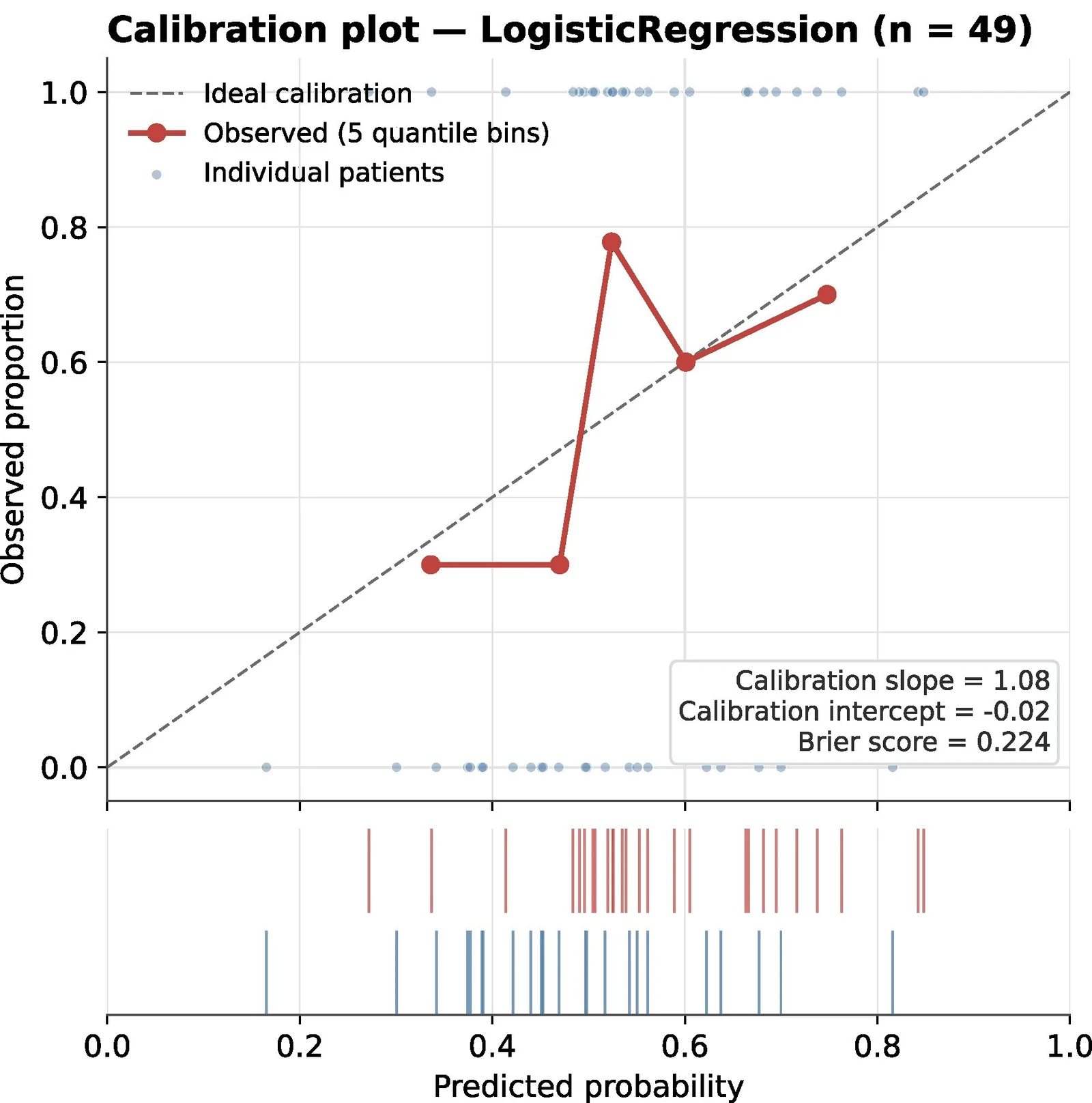
Calibration plot for the automated 3-feature logistic regression model (*n* = 49 patients). Predicted probabilities are plotted against observed outcomes for individual patients (light points) and for 5 quantile-based bins (line); the diagonal represents ideal calibration. The rug plot shows the distribution of predicted probabilities for patients with (upper ticks) and without (lower ticks) treatment response. Calibration slope, calibration intercept, and Brier score are annotated.

**Figure 9:**
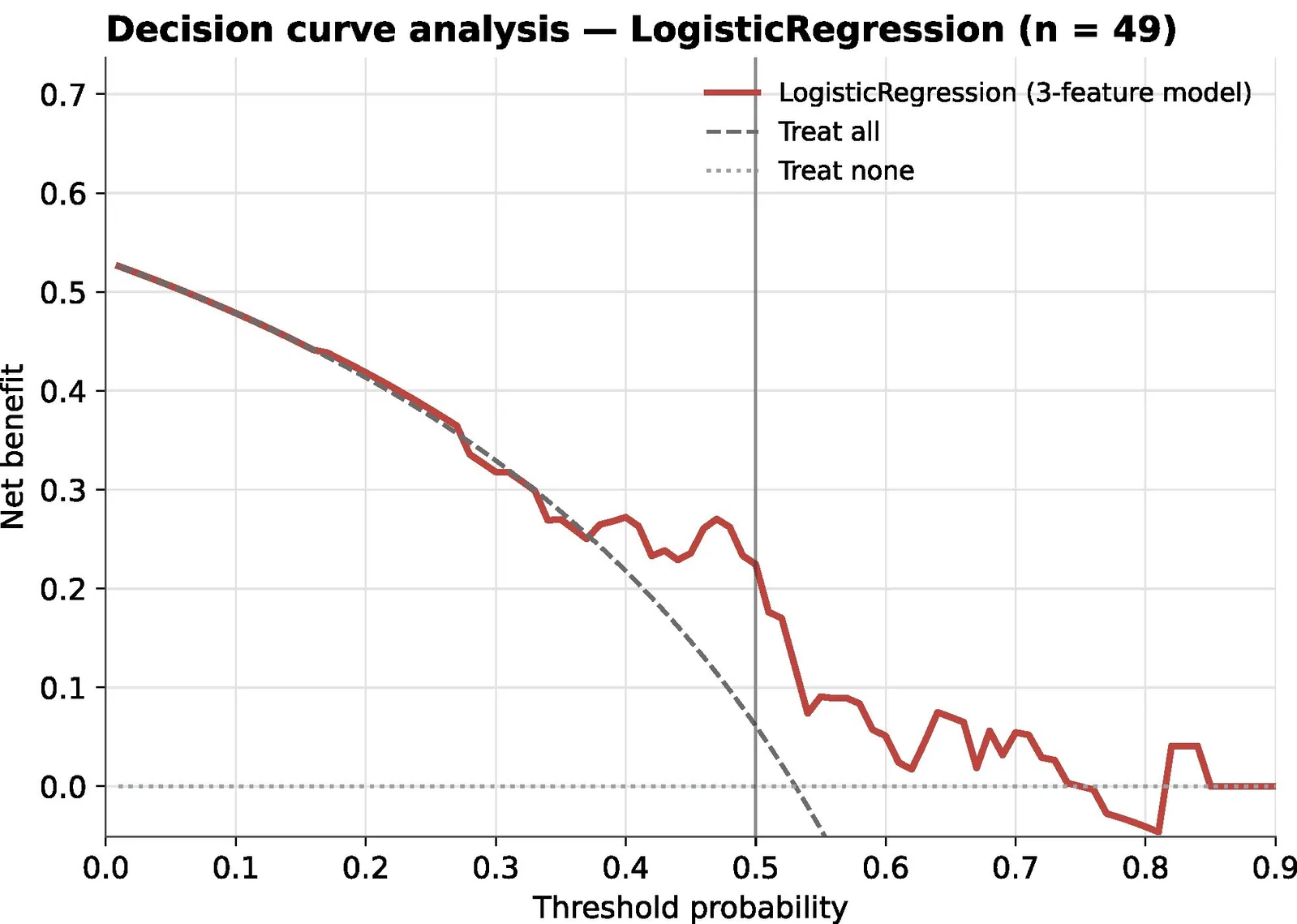
Decision curve analysis for the automated 3-feature logistic regression model (*n* = 49 patients). Net benefit of the model is plotted against threshold probability, alongside the default “treat-all” and “treat-none” strategies. The vertical line marks the 0.5 classification threshold used elsewhere in this study.

Calibration of the automated 3-feature LR model was good: calibration-in-the-large was 0.005, the calibration intercept was −0.023, the calibration slope was 1.082 (ideal values: 0, 0, and 1, respectively), and the Brier score was 0.224, indicating close agreement between predicted and observed risk despite the limited sample size (Fig. 8). DCA showed that the model provided greater net benefit than a “treat-all” strategy across essentially the entire clinically plausible range of threshold probabilities; at the 0.5 threshold, the model's net benefit (0.224) substantially exceeded that of “treat-all” (0.061) (Fig. 9).

The final model is reported in full as follows (Table 5):


$$\begin{array}{rl} & \log\;it(P[response])\\ & \;=5.079+0.0545\\ & \;\;\times [\log-sigma-3-0-mm-3D_{g\mathrm{lc}m_{ClusterShade}}]-0.00004\\ & \;\;\times [\log-sigma-2-0-mm-3D_{g\mathrm{lc}m_{ClusterShade}}]\\ & \;\;-1.090\times [square_{glrlm_{RunEntropy}}]; \end{array}$$

P[response]=1−(1+exp(−logit))

**Table 5:** Final model equation for the automated 3-feature logistic regression model, reported on the original (non-standardized) radiomic feature scale

Feature	Imputation median	Coefficient (original scale)
log-sigma-3-0-mm-3D_glcm_ClusterShade	2.290	+0.05450
log-sigma-2-0-mm-3D_glcm_ClusterShade	−4.430	−0.00004
square_glrlm_RunEntropy	4.933	−1.08965
Intercept	—	+5.07914

Missing feature values are imputed with the training-set median prior to evaluating the equation. This equation was verified to reproduce the fitted pipeline's predictions exactly and is provided to enable independent use and external validation of the model.

#### Feature importance and model interpretability

Native model coefficients, permutation importance, and SHAP values evaluated specifically for the primary 3-feature LR configuration converged on a singular conclusion: the predictive capacity of this optimized model was largely driven by a single candidate radiomic feature.

The regularization and model fitting assigned a near-zero impact to the third feature (log-sigma-2-0-mm-3D_glcm_ClusterShade), yielding a native coefficient of −0.002, which confirms its minimal contribution within this specific combination. Conversely, log-sigma-3-0-mm-3D_glcm_ClusterShade heavily dominated the 3-feature architecture with a prominent positive coefficient of 1.445. This primary biomarker was effectively complemented by square_glrlm_RunEntropy, which emerged as a crucial secondary predictor, exhibiting a substantial negative coefficient of −0.609.

This demonstrates that within the 3-feature configuration, therapeutic discrimination relies in practice on balancing coarse-scale textural asymmetry and cluster irregularity (at a spatial frequency of ∼3 mm) against the directional disorder and randomness of gray-level runs captured by the RunEntropy metric. Responders exhibited a significantly higher mean ClusterShade value and lower texture-based run entropy compared to nonresponders. LIME aggregate rules for this 3-feature model confirmed that extreme negative tails in ClusterShade accompanied by high RunEntropy values strongly directed predictions toward nonresponse, while positive ClusterShade values combined with lower run randomness steered the model toward therapeutic sensitivity.

### Methodological convergence and candidate feature stability

The convergence between cross-validation selection frequency, native model coefficients, permutation importance, and SHAP values suggests that log-sigma-3-0-mm-3D_glcm_ClusterShade captured a stable candidate signal within the internal validation framework. However, because this finding was derived from repeated cross-validation in a small cohort and was not tested in an independent clinical dataset, it should be interpreted as hypothesis-generating rather than as evidence of a validated imaging biomarker. The overarching dominance of GLCM texture features derived from multi-scale Laplacian of Gaussian filters confirms that the pipeline consistently captures coarse-scale textural asymmetry. This architectural consistency strongly supports the biological plausibility of tumor cluster irregularity as a stable, noninvasive surrogate marker of fgSRL therapeutic response, linked potentially to underlying somatostatin receptor sensitivity or distribution.

## Discussion

MRI remains the primary imaging modality for the diagnosis, characterization, and follow-up of pituitary adenomas. However, conventional radiological assessment is inherently subjective and may fail to capture the complex quantitative information embedded within medical images. In this context, advanced image analysis approaches, including deep learning–based segmentation and radiomics, offer the potential to extract reproducible and clinically meaningful information from routine MRI data.

In the present study, we first addressed the challenge of automatic tumor segmentation in a heterogeneous, multicenter setting. Using a public dataset, the SegResNet architecture achieved high performance (Dice = 0.835), reflecting the relative homogeneity of imaging protocols. However, when incorporating data from the ACROMICS/ACROFAST cohort, model performance decreased (Dice ranged around 0.76), highlighting the impact of real-world variability, including differences in acquisition protocols, scanner manufacturers, and image quality. Notably, the integration of a YOLO-based localization strategy improved segmentation performance, achieving a maximum Dice coefficient of 0.795 when combining Ce-T1WI and T2WI. These findings underscore the importance of robust pipeline design and multimodal input to mitigate domain shift and enhance generalization across institutions.

Despite these improvements, results did not reach the levels observed in homogeneous public datasets, reinforcing the notion that dataset heterogeneity remains a major limitation in the deployment of AI models in clinical practice. Furthermore, additional standardization strategies did not consistently improve results and, in some cases, even reduced them, suggesting that excessive normalization may remove relevant image information. This also indicates that modern architectures may partially compensate for intensity variability through internal normalization mechanisms.

Radiomics analysis was subsequently performed in a highly curated subgroup of 49 patients from the institutional cohort. This subgroup was restricted to patients treated with fgSRL exclusively, with suitable pretreatment Ce-T1WI and no uncertainty on expert neuroradiological review. This distinction is important: the larger 267-subject imaging dataset was used to develop and evaluate the automated detection and segmentation pipeline, whereas radiomics-based prediction of treatment response was performed only in patients with both appropriate baseline MRI and biochemical response data. Because only 49 of the 81 patients of the baseline cohort met all imaging and outcome requirements for radiomic analysis, we assessed the representativeness of the final cohort by comparing baseline characteristics between the 2 groups. No clinically meaningful differences were observed, suggesting that the final radiomic cohort remained representative of the original study population and providing no evidence of clinically relevant selection bias.

Under these stringent conditions, the automated leakage-controlled models showed fair-to-moderate discriminative performance. The automated 5-feature LR model achieved an OOF AUC of 0.712, while reducing the model to 3 automatically selected features modestly improved performance to an AUC of 0.729. In contrast, PCA-based dimensionality reduction yielded the poorest performance, suggesting that linear projection of the selected features did not preserve the discriminative structure required for response classification. These findings are consistent with the challenges of high-dimensional modeling in small clinical radiomics cohorts, while increasing the number of features may amplify variance without improving generalization.

Because the radiomic cohort was small and the initial feature space was high-dimensional, fold-wise feature selection was expected to show some instability. To explore whether the predictive signal was concentrated in a more reproducible subset of features, we performed a post hoc stability-informed sensitivity analysis using the most consistently selected features across the repeated cross-validation procedure. This fixed 3-feature LR model achieved higher apparent performance, with an OOF AUC of 0.798, balanced accuracy of 0.793, *F*1-score of 0.815, MCC of 0.590, and overall accuracy of 79.6%. However, because this fixed feature set was derived from the same internal resampling framework, this analysis should be interpreted as exploratory and hypothesis-generating rather than as an independent estimate of generalization performance.

The feature-level analyses suggested that the discriminative signal was largely driven by texture descriptors related to gray-level clustering and run-length heterogeneity. The most consistently selected candidate feature was log-sigma-3-0-mm-3D_glcm_ClusterShade, which reflects coarse-scale asymmetry and irregularity in gray-level co-occurrence patterns after Laplacian of Gaussian filtering. In the post hoc fixed 3-feature model, this feature was complemented by square_glrlm_RunEntropy, suggesting that response classification may depend on both cluster asymmetry and the randomness of gray-level runs within the adenoma. These findings are biologically plausible, as they may reflect intratumoral heterogeneity not captured by visual MRI assessment. Nevertheless, they should be considered candidate imaging markers pending validation in independent cohorts.

Importantly, all radiomics performance estimates in this study were derived from repeated cross-validation and OOF predictions within the same 49-patient cohort. Although this approach reduces overfitting compared with a single train-test split and allows more stable use of limited data, it does not replace validation in an independent external clinical cohort. The confidence intervals around model performance remain wide, and fold-to-fold variability reflects the statistical uncertainty inherent to small-sample radiomics studies. Therefore, the present findings should not be interpreted as evidence that the model is ready for clinical decision-making, but rather as support for further validation of a candidate radiomic signature.

To quantify this uncertainty directly, we performed a bootstrap-based stability analysis of the automated 3-feature model (1000 replicates), which confirmed that individual-level predictions are moderately unstable: the mean absolute prediction error across patients was 0.176, and bootstrap-derived models disagreed with the original model's classification for a given patient in 26.3% of replicates on average, rising to majority disagreement for 12.2% of patients (Fig. 6). We additionally quantified the optimism introduced by benchmarking 11 classifiers and selecting the best-performing one; this optimism was small (+0.0095 AUC points), and LR was the best-performing classifier in 68.0% of bootstrap replicates, suggesting that its selection was not primarily an artifact of the specific data partition used (Fig. 7). Notably, this stability analysis was performed on the automated 3-feature configuration (OOF AUC = 0.729) rather than on the higher-performing, fixed 3-feature model described above (OOF AUC = 0.798), which was derived post hoc from the same resampling framework; given its derivation, this fixed-feature configuration should be assumed to carry at least comparable, and plausibly greater, instability, reinforcing our interpretation of that result as exploratory and hypothesis-generating rather than confirmatory.

We also assessed model calibration and clinical utility directly. Calibration was good (calibration slope 1.082, calibration intercept −0.023, calibration-in-the-large 0.005), and DCA indicated that the model provides greater net benefit than a “treat-all” default strategy across the clinically plausible range of threshold probabilities (Figs. 8 and 9). We have also reported the final model as an explicit equation on the original feature scale (Table 5) to enable independent application and external validation, and completed a TRIPOD + AI checklist, provided as supplemental material (35).

When compared to previously reported radiomics studies in acromegaly, the performance observed here is lower. Prior works have reported AUC values typically ranging from 0.8 to 0.95 for endpoints such as tumor consistency, proliferative activity, granulation pattern, or radiotherapy response (14-16, 36, 37).

Several factors may explain this discrepancy. First, the prediction of response to fgSRL is inherently more complex, as it depends not only on tumor structure but also on molecular and receptor-level characteristics that may not be fully captured by imaging features alone. Second, the relatively small sample size used in this study limits model robustness and increases variability. Third, the strict inclusion criteria, while improving data quality, may have reduced sample diversity and statistical power.

In addition, our results are slightly lower than those reported in radiomic studies using T2WI for treatment response prediction, where AUC values around 0.80 to 0.85 have been described (38). This difference may reflect the fact that T2 signal intensity has a known biological association with tumor granulation pattern and somatostatin receptor expression, whereas Ce-T1WI-based radiomics may capture complementary but less directly related microarchitectural features.

From a clinical perspective, an AUC of 0.798 suggests that the radiomics model provides evidence of signal beyond chance, but its current performance and lack of independent validation are insufficient to support stand-alone clinical use. Instead, these findings support the role of radiomics as a complementary approach that may enhance predictive performance when integrated with clinical, biochemical, and molecular data.

From a clinical perspective, the relevance of radiomics lies not in image analysis itself, but in its potential to transform routinely acquired MRI into a quantitative biomarker capable of supporting therapeutic decision-making. Such an approach could reduce the current trial-and-error strategy and facilitate earlier implementation of precision medicine in acromegaly. Our study should be interpreted within the current paradigm of precision medicine in acromegaly, where imaging, functional and molecular biomarkers are increasingly being incorporated into treatment algorithms rather than replacing clinical judgment.

Importantly, the combination of robust automatic segmentation—such as the one developed in this study—with radiomics pipelines represents a key step toward scalable and reproducible quantitative imaging biomarkers. At this stage, the identified ClusterShade feature should be viewed as a candidate imaging marker for future validation, ideally in a larger multicenter cohort with harmonized MRI acquisition, predefined preprocessing, and comparison against established clinical and radiological predictors.

Overall, this work highlights both the potential and the current limitations of AI-driven analysis in acromegaly. While automatic segmentation approaches can achieve robust performance even in heterogeneous datasets, radiomics-based prediction of treatment response remains challenging. Further methodological refinement, larger multicenter cohorts, and integration into multimodal predictive frameworks are required to achieve clinically useful performance as a single biomarker.

By extracting high-dimensional quantitative features from MRI datasets and refining them into parsimonious descriptors, it is expectable that subsequent research using this methodology of imaging characterization may enable a more comprehensive characterization of tumor phenotype. If confirmed in adequately powered external validation studies, this approach could contribute to precision medicine strategies in acromegaly. As mentioned, growing evidence supports its utility in differentiating pituitary tumor subtypes, predicting tumor consistency and invasiveness, thereby enhancing valuable information at the preoperative stage (36). Nevertheless, the clinical implementation of radiomics in acromegaly, as shown in the present study, remains so far challenging. Key limitations include concerns regarding the reproducibility and robustness of imaging features, as well as the limited number of MRI-based radiomics studies specifically addressing somatotropinomas. Despite these limitations, the data so far published give support to the concept that quantitative imaging can capture clinically relevant tumor heterogeneity. In this regard, prediction of tumor consistency in acromegaly, as recently published (15), demonstrating that texture- and shape-based features could distinguish soft from firm adenomas and thereby inform surgical planning, is very relevant from the clinical point of view. In the same direction, a machine-learning multiparametric radiomics signature has been demonstrated to predict radiotherapeutic response, showing that baseline MRI features combined with clinical data could noninvasively stratify patients by likelihood of remission after pituitary radiotherapy (14). Extending beyond mechanical and treatment response endpoints, a subsequent multicenter investigation has built a radiomics-based nomogram to estimate Ki-67 index, indicating that proliferative activity can also be inferred from quantitative MRI patterns (16). Finally, another study applied radiomics to classify densely vs sparsely granulated GH-secreting pituitary adenomas, providing a noninvasive surrogate of granulation pattern that is closely related to somatostatin receptor ligand responsiveness (37). Collectively, these 5 studies, including the present work, suggest that radiomics may interrogate multiple biological and therapeutic dimensions of acromegaly, such as tumor consistency, proliferative activity, granulation pattern, and both medical treatment and radiotherapy outcomes, and thus offer a promising framework to develop predictive models for treatment response in acromegaly.

## ACROMICS/ACROFAST research group

Manel Puig-Domingo, Elena Valassi, Montserrat Marques-Pamies, Silvia Martínez-Couselo, Cristina Carrato, Joan Gil, Paula de Pedro, Federico Vázquez, Mireia Jordà (Hospital i Institut de Recerca Germans Trias, Badalona), Betina Biagetti, Silvana Sarria (Hospital Vall d’Hebrón, Barcelona), Olga Giménez-Palop, Anna Aulinas, Susan M. Webb (Hospital de Sant Pau, Barcelona), Marta Hernández, Jordi Ferri (Hospital Clínico de Valencia), Rocío Villar-Taibo, Ignacio Bernabéu (Hospital Universitario de Santiago de Compostela), Marta Araujo-Castro, Eider Pascual (Hospital Ramón y Cajal, Madrid), Concepción Blanco (Hospital de Alcalá de Henares), Inmaculada Simón, Roxana Zavala, (Hospital) Joan XXIII, Tarragona), Andreu Simó-Servat (Hospital Universitari Mutua de Terrassa), Gemma Xifra (Hospital Universitari Josep Trueta, Girona), Isabel Pavón, José Antonio Rosado (Hospital Universitario de Getafe), Rogelio Garcìa-Centeno (Hospital Gregorio Marañón, Madrid), Felicia A Hanzu, Mireia Mora (Hospital Clínic, Barcelona), Nuria Vilarrasa, Fernando Guerrero (Hospital Universitari de Bellvitge, Barcelona), Soledad Librizzi, María Calatayud (Hospital 12 de Octubre, Madrid), Paz de Miguel (Hospital Clínico de San Carlos, Madrid), Cristina Alvarez-Escola (Hospital Univeristario La Paz, Madrid), Antonio Picó (Hospital Universitario de Alicante), Carmen Fajardo-Montañana (Hospital Universitario de La Ribera, València) Eva Benegas, Alfonso Soto (Hospital Virgen del Rocío, Sevilla), María-Angeles Galvez, Raúl Luque (Hospital Universitario Reina Sofia e Instituto de Investigación Maimónides, Córdoba), Cristina Lama, Edelmiro Menéndez, Esteban Delgado (Hospital central de Asturias), Guillermo Serra (Hospital Universitari Son Espases, palma de Mallorca), Miguel Sampedro, and Mónica Marazuela (Hospital Universitario La Princesa, Madrid).

## Contributors

A.R.J. and M.P.D. performed the data collection and data analysis; R.M.E. and A.I.G. performed the segmentation under the supervision of G.M.R., and P.P. performed the validation of the segmented images. All Images were provided by the investigators of the ACROFAST/ACROMICS Research group, in particular by M.A.-C., M.M., F.R., B.B., E.V., and F.V. All the radiomic analyses were performed by M.L.M. and J.A.A. under the supervision of M.I.V. M.P.-D., S.R.J., R.M.E., J.A.A., and G.M.R. wrote the manuscript which was reviewed by all the authors. All the authors declare that they have no conflict of interest in relation to this work.

## Data Availability

Some or all datasets generated during and/or analyzed during the current study are not publicly available but are available from the corresponding author on reasonable request.
