# Correction to: “MRI-Based Radiomics Prediction of Somatostatin Receptor Ligand Response in Acromegaly: An Automated Pipeline Study”

**DOI:** 10.1210/jendso/bvag231

**Published:** 2026-10-12

In the above-named article by Ruiz S, Mateu-Estivill R, Izquierdo-Gómez A, López-Máñez M, Alzate JA, Monté-Rubio G, de la Iglesia-Vayá M, Araujo-Castro M, Biagetti B, Sarria S, Vázquez F, Romero F, Valassi E, Navas V, Marazuela M, Puyalto P, and Puig Domingo M on behalf of the ACROMICS/ACROFAST Research group (J Endo Society. 2026; 10(10): bvag198; doi: 10.1210/jendso/bvag198), there was an error in the author list.

In the originally published article, author Silvana Sarria-Estrada's name was shown as Silvana Sarria. In the corrected article, this author's name has been corrected to Silvana Sarria-Estrada.

The article has been corrected online.

doi: 10.1210/jendso/bvag198

